# State Observer for Linear Systems with Explicit Constraints: Orthogonal Decomposition Method

**DOI:** 10.3390/s21186312

**Published:** 2021-09-21

**Authors:** Sergei Savin, Oleg Balakhnov, Ramil Khusainov, Alexandr Klimchik

**Affiliations:** Center for Technologies in Robotics and Mechatronics Components, Innopolis University, Innopolis 420500, Russia; o.balakhnov@innopolis.university (O.B.); r.khusainov@innopolis.ru (R.K.); a.klimchik@innopolis.ru (A.K.)

**Keywords:** state observer, dynamic output feedback, explicit constraints, walking robots

## Abstract

In this paper, an orthogonal decomposition-based state observer for systems with explicit constraints is proposed. State observers have been an integral part of robotic systems, reflecting the practicality and effectiveness of the dynamic state feedback control, but the same methods are lacking for the systems with explicit mechanical constraints, where observer designs have been proposed only for special cases of such systems, with relatively restrictive assumptions. This work aims to provide an observer design framework for a general case linear time-invariant system with explicit constraints, by finding lower-dimensional subspaces in the state space, where the system is observable while giving sufficient information for both feedback and feed-forward control. We show that the proposed formulation recovers minimal coordinate representation when it is sufficient for the control law generation and retains non-minimal coordinates when those are required for the feed-forward control law. The proposed observer is tested on a flywheel inverted pendulum and on a quadruped robot Unitree A1.

## 1. Introduction

State estimation is needed when values of some of the state variables required by the control law are not directly available from the measurements. This can be the result of the lack of sensors, measurement noise, or be the result of the chosen parametrization of the system. In a typical scenario, control law requires full state information, while only some of the states are directly measured. This problem has been addressed in great detail, leading to solutions in the form of, among others, optimal Dynamic Output Feedback, Luenberger State Observers, and Kalman Filters. The related theory has been well developed for linear dynamical systems; however, it has also been extended and actively applied to nonlinear dynamical systems, in particular to mechanical systems and robots, often with the use of local linearization of their nonlinear dynamics.

While many mechanical systems and robots can be effectively described by systems of ordinary differential equations, making it possible to use the first term in Taylor expansion of their dynamics to apply aforementioned linear state estimation methods, some are better described as differential-algebraic equations (DAE); these include systems with explicit mechanical constraints [1]. In particular, walking robots, robot arms in contact with the environment, and many other similar systems are better described with a specific conventional set of coordinates, which can be non-minimal; for walking robots, this description is called floating-base dynamics, combining joint coordinates and the position and orientation of the robot’s “base”, which is usually a robot’s trunk [2,3,4]. There are a number of reasons why this description can be favored: it allows for maintaining the structure of the equations while contacts are being acquired and released, it explicitly includes contact forces, allowing to place them under additional constraints, such as a friction cone, and, in some cases, it is simply difficult to find a singularity-free minimal coordinate representation for multi-body dynamics. However, dynamics represented as a DAE poses a problem when classical control and state estimation methods are to be applied.

Over the last 40 years, there has been a continuous research effort in establishing control and estimation methods, suitable for systems with explicit constraints. Theory and methods have been well developed for a general case when systems with explicit constraints were presented in descriptor form [5,6,7]. Separately, notable progress has been seen in solving an inverse dynamics problem for the second-order systems with constraints [3,4]. Both of those will be discussed in more detail in the next section. A few types of stabilizing control have been proposed: based on second-order error dynamics with suitable projections, and on projecting local linearization of the dynamics equations into the null space of the constraints matrix, allowing for formulating linear-quadratic regulator for the constrained system [8,9]. However, an equivalent observer design (based on orthogonal decomposition) so far has not been proposed. As will be discussed in the following sections, linear dynamics of the systems with explicit constraints naturally takes affine form, due to the fact that part of the state is constrained to be constant. With that in mind, it is important not only to design stabilizing feedback control but also the appropriate feed-forward component, to account for affine terms in the system dynamics.

Additionally, constraints introduce closed kinematics chains, making the systems over-actuated; on the other hand, in the absence of constraints systems such as floating-base walking robots are underactuated and not controllable. This creates additional challenges regarding state estimation, as some of the states in the floating base coordinates are neither observable nor do they influence the dynamics of the system. Moreover, the structure of the dynamics of mechanical systems makes the same variables appear both in the state and in its time derivative (this happens with generalized velocities in Lagrange dynamics or manipulator equations when those are expressed in state-space form). This information can be lost if the constraints are placed on the state derivatives only, as was done in [8]; if accounted for, it additionally simplifies the state estimation task, as we will show in Section 6.

The main contributions of this paper are as follows:

1. we propose a state observer that converges to the true values of the state for linear dynamical systems with explicit mechanical constraints;
2. we formulate a lower-dimensional state observer, able to take advantage of the constraints placed explicitly on the state variables, which is a natural scenario for mechanical systems; thus, the obtained observer has demonstrably lower requirements with respect to the available measured outputs, without compromising the control law design;
3. we propose an additional change of basis, based on the intersection of the row space of the state matrix for static states, and the state space of the previously mentioned observer, leading to an observer with a lower-dimensional state, able to recover minimal coordinate representation, as we demonstrate in Section 9.

The rest of the paper is organized as follows: Section 2 gives an overview of the state of the art and expands on where the proposed method stands relative to the previous ones, Section 3 gives a brief description of the materials and methods used, Section 4 gives a mathematical formulation of the problem and covers preliminaries, Section 5 proposes constrained observer design, Section 6 gives a lower-dimensional version of the observer, overcoming the limitations of the constraint representation, Section 7 proposes an even lower-dimensional version, excluding the static states that do not play in the feedback or feed-forward components of the control law, Section 8 discusses gain design for the proposed observer, Section 9, Section 10 and Section 11 discuss experimental and numerical case-studies: flywheel inverted pendulum, quadruped robot Unitree A1, and a flat quadruped moving in the sagittal plane, Section 12 demonstrates a numerical comparison study of the proposed Observer and an Extended Kalman Filter, and Section 13 discusses the application of the proposed method in control and sensor distribution design for mobile robots.

## 2. State of the Art

### 2.1. Orthogonal Projection and Decomposition Methods for Systems with Explicit Constraints

A state estimation method proposed in this paper is based on orthogonal projections. There is a number of existing orthogonal projection and decomposition-based methods developed for systems with explicit constraints, which we review in this subsection. The results closest to the one proposed in the paper are found in papers on control of mechanical systems with explicit constraints, so we focus our discussion on those.

Models of mechanical systems with explicit constraints are often presented as differential-algebraic equations, which prevents direct application of methods, designed for ordinary differential equations (ODE). This motivated a group of work, aimed at replacing the DAE description of the dynamics with an equivalent ODE. In [10], a procedure was proposed for simulation of mechanical systems with explicit constraints, which establishes a connection with earlier works in the field of numerical methods for DAE, such as [11] and others, which tackled a very similar problem. In [10,12], as well as in later works [3,4,13], orthogonal projections have been used. In [10,12], a number of new equivalent models in non-minimal coordinates were proposed, while, in [3,4,13], following earlier research [14,15], the focus was on control law design. However, as was demonstrated in [4], a number of the independently developed projection-based control methods yield the same (up to torque redundancy resolution) control laws.

Another family of projection-based methods have been designed for inverse dynamics problem: the problem of finding such control input that produces desired acceleration for a mechanical system [3,4,16]. Alternative formulations have been proposed; in [17], the DAEs were solved directly, and, in [18,19,20] and others, convex optimization has been used to solve the problem, taking into account contact models (such as friction cone). We should note that the presented methods assume partial or complete knowledge of the state of the system.

The last family of projection-based methods we mention here are schemas for solving the linear-quadratic regulator (LQR) problem for mechanical systems with explicit constraints. The solution to this problem was originally proposed in [8,9] and in a different form in [21]. The method presented in [8] takes advantage of the known structure of the constraint matrix to project the locally linearized dynamics into its null space, resulting in a local linear model in minimal coordinates. With that, LQR can be formulated and optimal feedback control gains can be found. We should note that the method is motivated by practical problems, such as control of bipedal robots, which are naturally nonlinear. Therefore, the methods proposed in [8,9] take locally linearized models, but then use them to design infinite-horizon LQR or a time-variant LQR where the model is obtained by linearization along the nominal trajectory. While guarantees of stability of the nonlinear closed-loop dynamics and optimality of the proposed control law are a subject of further investigation, the practical applicability of the methods has been demonstrated in hardware experiments and its simplicity advocates for its use. Present work has a similar motivation, and the new observer designs we propose for the systems with explicit constraints can be used in a similar way. The same as control design presented in [8], our state estimation method takes advantage of the orthogonal projections, but, in order to construct the state observer, we consider both the null space and the row space components of the linear dynamics and take advantage of the linear relations between components of the state and the state derivative, which allow for writing constraints placed on the system’s state directly and exploit them in the observer design.

### 2.2. State Estimation for Systems with Explicit Constraints

Systems with explicit constraints can be represented as so-called descriptor systems, which can be thought of as a form of a DAE or as a mix of static and dynamic equations with control inputs [5]. Concerning state estimation, the descriptor system theory presented a wide range of results: state observers for general-case linear descriptor systems were proposed and conditions for their existence were established [6,7,22,23,24,25], extensions for some classes on nonlinear descriptor systems were proposed [26,27] and adaptive and robust observers were designed [28,29], among other contributions. Additionally, it was demonstrated that mechanical systems with constraints can be written in the descriptor form [1] and Luenberger observers for such systems were proposed [30,31]. This body of work might be unduly overlooked in modern legged robotics and similar fields. However, one of the limitations of the methods developed for descriptor systems is the aim to estimate the whole descriptor state, which in the case of mechanical systems includes position and velocity variables, as well as aggregate reaction forces [1]; it is easy to find examples where such a system is not observable, while estimation of only a subset of the state variables is sufficient for stabilizing feedback control design (we provide one such example in the paper). Our contribution compared to results in descriptor systems theory is the systematic way to produce a lower-dimensional state observer based on orthogonal projections, which can be seen as automatically recovering a minimal representation of the static component on the descriptor state.

We continue by briefly discussing state-of-the-art techniques in state estimation of walking robots, since walking robot models are among the most prominent examples of systems with explicit constraints. In [32], an observer design for a flat bipedal robot is considered, and reaction forces multiplied by constraints Jacobian are viewed as an additive uncertain term in the dynamics model, allowing for freeing the problem formulation from explicit constraints. In [33], an observer is designed to estimate the state of the so-called ’cart on the table’ model, which represents a simplified model of the motion of the center of mass of the walking robot. In [34], the motion of a humanoid robot was divided into phases based on the contact interaction scenario, and then the unknown reaction forces were expressed out of the dynamics equation, leading to a formulation with no explicit constraints, which allowed the application of the classic Luenberger observer. A similar approach is shown in [35]. In [36], the same was done but an Extended Kalman Filter was used. In [37], the sliding-mode observer was used, with no explicit constraints in the observer formulation.

Overall, one of the more prevailing approaches is the use of extended Extended Kalman Filter to estimate the state of the robot, taking advantage of the information about the gait and expected nature of the contact interaction with the environments and the kinematic structure of the robot, while making some assumptions about mentioned contact interactions and the availability of specific sensors [38,39,40,41]. These methods have been successfully used for legged robots in a number of experiments and practical tasks. In relation to the method proposed in this paper, we can see them as effective use of platform-specific properties to build a highly effective estimator, whereas the proposed method enjoys the advantage of being general, potentially requiring less work to be adapted to a new platform. This relation is similar to the one between linear-quadratic regulators and platform-specific Model-Predictive Control (MPC) algorithms developed for specific types of legged robots.

Finally, we note that, in some cases, the walking robot dynamics can be formulated in a hybrid constraint-free form, which allows the design of hybrid dynamics state observers and disturbance observers [42,43]. However, their use is limited to the cases when a hybrid description of the system’s dynamics is available.

**Figure 1:**
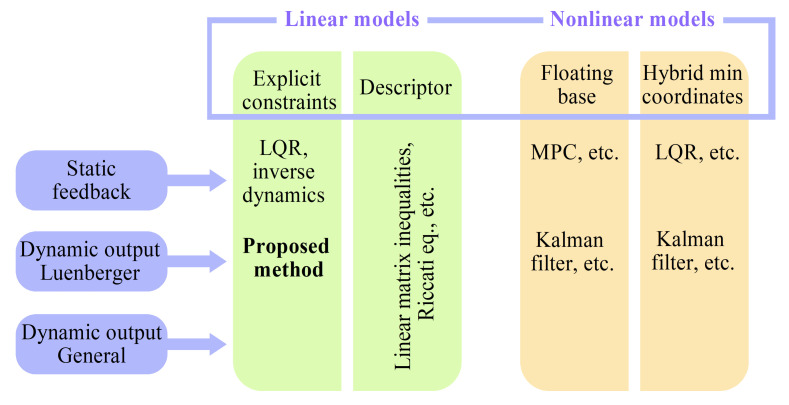
Diagram of control and state estimation methods for systems with explicit constraints.

Figure 1 presents a diagram, sorting the aforementioned multitude of methods with respect to the plant model types they use (linear: with explicit constraints and in descriptor form, and nonlinear: in floating-base form and in hybrid dynamics form with minimal coordinates), and the type of feedback control they implement: full state feedback (static feedback) and dynamic output feedback (in Luenberger form and in general form).

Analyzing the diagram, we note that, while feedback control design is well-developed for hybrid systems in minimal coordinates, the corresponding hybrid dynamics models are not easy to work with for high-dimensional systems with numerous and varied contact interactions, such as quadrupeds and humanoid robots. Using a nonlinear floating-base description of the dynamics leads to non-minimal coordinates, and allows the use of a number of platform-specific solutions. A more general framework has been built for linear dynamical systems expressed in descriptor form, where some solutions have been found for both static and dynamic output feedback, subject to conditions on admissibility and observability. Depending on the formulation, the descriptor vector includes both state variables and reaction force variables; it can be shown that, for some cases, a lower-dimensional observer would be sufficient for control design, whereas observing the full state might not be possible (as demonstrated in Section 9). This is where linear controller and observer designs based on subspace projections have an advantage. The method proposed in this paper gives an observer design based on null and row space projections for the constraint matrix, taking additional advantage of the constraints placed directly on the state variables and of the analysis of the effect static states have on the dynamics of the system, automatically excluding those that have none from the observer state.

The proposed method can be seen as a combination of a Luenberger observer for the space of non-static states and a disturbance observer for the rest. If the control input that turns the system into a node is exactly known (via Inverse dynamics, for example), the observation of the static component of the state might not be needed. However, as we mentioned before, algorithms that solve this problem usually assume that information about the full state of the robot is available [3,4]. Thus, the proposed observer fills the gap in the family of methods for systems with explicit constraints based on orthogonal projections.

## 3. Materials and Methods

This paper presents a new type of state observer based on the orthogonal projections of the system’s dynamics. The bulk of the work is done via analytic derivations, verified in simulations and experimental studies. For verification, a table-top flywheel inverted pendulum was used, described in Section 9. Details of the experiment, measurements, and the model of the robot are presented in the section. For further analysis, a quadruped robot Unitree A1 was used; the parameters of this robot are publicly available.

## 4. Problem Statement

### 4.1. Dynamics with Explicit Constraints: Model

**Figure 2:**
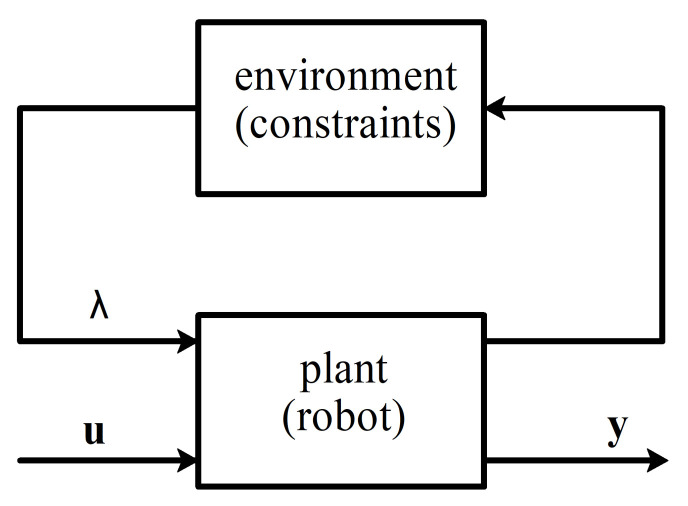
System with explicit constraints as a block diagram.

A linear time-invariant dynamical system with explicit constraints can be presented as follows:

(1) $$\left\{\begin{array}{c} \dot{x}=Ax+Bu+F\lambda \\ G\dot{x}=0\\ y=Cx, \end{array}\right.$$

where $x\in R^{n}$, $u\in R^{m}$ and $y\in R^{h}$ are state, control input, and measured output, A, B, and C are state, control, and observation matrices, respectively, F and G are constraint Jacobian and constraint matrix and $\lambda \in R^{k}$ are reaction forces (or Lagrange multipliers). From the given formulation, it follows that the rate of change for the state of the system lies in the subspace of $R^{n}$. We can treat λ as input generated by the environment, acting on the plant, which would allow us to use linear-fractional transformation on it (see Figure 2). Expressing out λ leads to the following matrix representation:

(2) $$\dot{x}=\left(I-F{\left(GF\right)}^{-1}G\right)\left(Ax+Bu\right)$$

System (2) represents dynamics (5). Note that, while (5) has explicit DAE form, (2) appears to be an ODE; however, the values of $\dot{x}$ still belong to the null space of constraint matrix G; the transformation (2) only rids the equations of the reaction forces λ. We introduce short-hand notation for the matrices of this system:

(3) $$\begin{array}{cc} & A_{c}=\left(I-F{\left(GF\right)}^{-1}G\right)A \end{array}$$

(4) $$\begin{array}{cc} & B_{c}=\left(I-F{\left(GF\right)}^{-1}G\right)B. \end{array}$$

where $A_{c}$ and $B_{c}$ are state and control matrices of the system (2), which now takes the form:

(5) $$\left\{\begin{array}{c} \dot{x}=A_{c}x+B_{c}u\\ G\dot{x}=0\\ y=Cx, \end{array}\right.$$

With that, we can use some of the standard results known from the literature on systems with explicit constraints, such as the linear feedback method from [8] that we will introduce later in this section. Note that matrices $A_{c}$ and $B_{c}$ can also be obtained as a linearization of nonlinear dynamics, solved for the higher derivatives, as illustrated by the next example.

**Example** **1.** *Consider a mechanical system with explicit constraints:*

$$\left\{\begin{array}{c} H_{n}\ddot{q}+C_{n}\dot{q}+g_{n}=T_{n}u+F_{n}^{⊤}\lambda ,\\ F_{n}\ddot{q}+{\dot{F}}_{n}\dot{q}=0 \end{array}\right.$$

*where q are generalized coordinates, $H_{n}$, $C_{n}$, and $T_{n}$ are generalized inertia, Coriolis and control matrices, $g_{n}$ is the generalized gravity vector and $F_{n}$ is a constraint Jacobian; all mentioned quantities are nonlinear functions of q. We can solve for $\ddot{q}$:*

$$\ddot{q}=f_{n}\left(q,\dot{q},u\right)=\left(I-M_{n}F_{n}\right)H_{n}^{-1}\left(T_{n}u-C_{n}\dot{q}-g_{n}\right)+M_{n}{\dot{F}}_{n}\dot{q},$$

*where $M_{n}=H_{n}^{-1}F_{n}^{⊤}{\left(F_{n}H_{n}^{-1}F_{n}^{⊤}\right)}^{-1}$. Defining state as $x={\left[q^{⊤},\;\;{\dot{q}}^{⊤}\right]}^{⊤}$, matrices $A_{c}$, $B_{c}$ and G for this system can be found as:*

(6) $$A_{c}=\frac{\partial f_{n}}{\partial x},\;\;B_{c}=\frac{\partial f_{n}}{\partial u},\;\;G=\left[\begin{array}{cc} F_{n} & 0\\ {\dot{F}}_{n} & F_{n} \end{array}\right].$$

As we can see from the example, the expression (5) can be generated directly from the nonlinear dynamics, bypassing the form (1). Other approaches to generate expression (5) are shown in [9,21].

**Remark** **1.** *From (3) and (4), or from (6), it is easy to observe that the first equation in the system (5) will always satisfy constraints $G\dot{x}=0$; in fact, there are no reaction forces to enforce these constraints. That begs the conclusion that explicit constraints can be dropped out of the expression (5). However, these explicit constraints contain information about the subspace to which the state derivative $\dot{x}$ is bound, which is instrumental in designing control law for the system with the use of appropriate projections, as will be shown in the next sections.*

### 4.2. Dynamics with Explicit Constraints: Control

Let us denote $N=\mathrm{null}\left(G\right)$ and $R=\mathrm{col}\left(G^{⊤}\right)$, where $\mathrm{null}\left(\cdot \right)$ and $\mathrm{col}\left(\cdot \right)$ are operators, returning orthonormal bases in, respectively, null space and column space of the input matrix. Then, we can represent $\dot{x}$ and x in terms of the bases N and R:

(7) $$\begin{array}{cc} & \dot{x}=N\dot{z} \end{array}$$

(8) $$\begin{array}{cc} & x=Nz+R\zeta , \end{array}$$

where $z=N^{⊤}x\in R^{n_{z}}$ and $\zeta =R^{⊤}x\in R^{n_{\zeta }}$ are coordinate representation of the projection of x onto the span of N and R. Vector ζ represents states (expressed in basis R) that are static: their values cannot change under given constraints; z represents linearly independent non-static states (expressed in basis N).

Following [8,9], we project (5) into the span of N, arriving at the following *zero dynamics* formulation:

(9) $$\dot{z}=N^{⊤}A_{c}Nz+N^{⊤}B_{c}u+N^{⊤}A_{c}R\zeta .$$

We note that, in [8,9], the constant component $N^{⊤}A_{c}R\zeta$ is not mentioned explicitly. It can be seen as one of the many sources of constant terms in affine dynamics, affecting the steady-state solution, but not the stability of the system. However, as we will demonstrate in the rest of the paper, a systematic approach to identifying both constant and time-varying terms in the system dynamics leads to effective state estimation and control algorithms.

In [8,9], it is proposed to design feedback control for system (9) by stabilizing the following closed-loop dynamics by finding control gains $K_{z}$:

(10) $$N^{⊤}A_{c}N-N^{⊤}B_{c}K_{z}\in H$$

where H is the set of all matrices whose all eigenvalues have negative real parts; M∈H means that the matrix M is Hurwitz. As an example, control gains $K_{z}$ can be found by solving the LQR problem.

Resulting control law takes the form $u=-K_{z}z=-K_{z}Nx$, leading to closed loop dynamics $\dot{z}=N^{⊤}\left(A_{c}-B_{c}K_{z}\right)Nz+N^{⊤}A_{c}R\zeta$. This makes it clear that state matrix $A_{c}-B_{c}K_{z}$ does not have to be stable, only its projection onto the span of N does. It is also clear that the steady-state solution will lie at the origin only if $N^{⊤}A_{c}R\zeta =0$.

We can propose an affine control law that stabilizes the origin: $u=-K_{z}z+u_{\zeta }$. In order for $\dot{z}=0$ when z=0, the following needs to hold:

(11) $$N^{⊤}B_{c}u_{\zeta }=-N^{⊤}A_{c}R\zeta ,$$

which means that the origin can become a node as long as vector $N^{⊤}A_{c}R\zeta$ lies in the column space of $N^{⊤}B_{c}$, which can be represented as the following condition:

(12) $$\left(I-N^{⊤}B_{c}{\left(N^{⊤}B_{c}\right)}^{+}\right)N^{⊤}A_{c}R\zeta =0,$$

where $I\in R^{n\times n}$ is an identity matrix, and ${\left(\cdot \right)}^{+}$ is a Moore–Penrose pseudo-inverse. If condition (12) holds, the value of $u_{\zeta }$ can be calculated as follows:

(13) $$u_{\zeta }=-{\left(N^{⊤}B_{c}\right)}^{+}N^{⊤}A_{c}R\zeta ,$$

Denoting $K_{\zeta }={\left(N^{⊤}B_{c}\right)}^{+}N^{⊤}A_{c}R$, we get control law in the form:

(14) $$u=-K_{z}z-K_{\zeta }\zeta .$$

We would like to note here that variable ζ comes into the dynamics equations only as input to the operator R. This will be used in the following sections when the pair ζ, R will be replaced with lower-dimensional projections, without changing the structure of the equations.

As was said previously, the use of state estimation is justified by the need to use the full state in the control law, as was exemplified in this section. The next section provides the proposed formulation of a state estimator for constrained LTI systems.

## 5. State Estimation for LTI Systems with Explicit Constraints

In this section, we propose a method for estimating the state of an LTI system with explicit constraints. We start by observing that, in previously introduced notation $\dot{\zeta }=0$, and therefore we can write the following description of the dynamics:

(15) $$\left\{\begin{array}{c} \left[\begin{array}{c} \dot{z}\\ \dot{\zeta } \end{array}\right]=\left[\begin{array}{cc} N^{⊤}A_{c}N & N^{⊤}A_{c}R\\ 0 & 0 \end{array}\right]\left[\begin{array}{c} z\\ \zeta \end{array}\right]+\left[\begin{array}{c} N^{⊤}B_{c}u\\ 0 \end{array}\right]\\ y=C\left[\begin{array}{cc} N & R \end{array}\right]\left[\begin{array}{c} z\\ \zeta \end{array}\right] \end{array}\right.$$

We will refer to this representation of the dynamics as *subspace representation*. Using this representation, we can propose the following state observer:

(16) $$\left[\begin{array}{c} \dot{\hat{z}}\\ \dot{\hat{\zeta }} \end{array}\right]=\left[\begin{array}{cc} N^{⊤}A_{c}N & N^{⊤}A_{c}R\\ 0 & 0 \end{array}\right]\left[\begin{array}{c} \hat{z}\\ \hat{\zeta } \end{array}\right]+\left[\begin{array}{c} N^{⊤}B_{c}u\\ 0 \end{array}\right]+L\left(y-C\left[\begin{array}{cc} N & R \end{array}\right]\left[\begin{array}{c} \hat{z}\\ \hat{\zeta } \end{array}\right]\right),$$

where $\hat{z}$ and $\hat{\zeta }$ are estimates of z and ζ.

As long as the system in the proposed subspace representation is observable, we can ensure the stability of the resulting system by choosing correct observer gains L. We stress the fact that chosen representation does play a role, as we will demonstrate in the following sections. The correct choice of observer gains L is given by the following theorem:

**Theorem** **1.** *Observer (16) and dynamics (9), with control law $u=-K_{z}\hat{z}-K_{\zeta }\hat{\zeta }$ form a stable system as long as (12) holds, control gains $K_{z}$ are chosen to satisfy condition (10) and observer gains are chosen to satisfy the next condition:*

(17) $$\left[\begin{array}{c} N^{⊤}\\ R^{⊤} \end{array}\right]\left(\left[\begin{array}{c} A_{c}^{⊤}N\\ 0 \end{array}\right]-C^{⊤}L^{⊤}\right)\in H$$

Note that condition (17) is written dual form, making the observer gains appear on the right side of the observation matrix, allowing for finding stabilizing gains via pole placement or LQR, as will be shown in Section 8.2.

**Proof** **of Theorem 1.** Defining observation error $e=\left[\begin{array}{c} z-\hat{z}\\ \zeta -\hat{\zeta } \end{array}\right]$ and subtracting (16) from (15), we get observer error dynamics:

(18) $$\dot{e}=\left(\left[\begin{array}{cc} N^{⊤}A_{c}N & N^{⊤}A_{c}R\\ 0 & 0 \end{array}\right]-LC\left[\begin{array}{cc} N & R \end{array}\right]\right)e$$

Substituting control law into (9), we find state dynamics as:

(19) $$\dot{z}=\left(N^{⊤}A_{c}N-N^{⊤}B_{c}K_{z}\right)z+N^{⊤}B_{c}Ke+\left(N^{⊤}A_{c}R-N^{⊤}B_{c}K_{\zeta }\right)\zeta .$$

where $K=\left[\begin{array}{cc} K_{z} & K_{\zeta } \end{array}\right]$. With that, we can write the combined state and observer dynamics:

(20) $$\left[\begin{array}{c} \dot{z}\\ \dot{e} \end{array}\right]=\left[\begin{array}{cc} \left(N^{⊤}A_{c}N-N^{⊤}B_{c}K_{z}\right) & N^{⊤}B_{c}K\\ 0 & ({\bar{N}}^{⊤}A_{c}-LC)E \end{array}\right]\left[\begin{array}{c} z\\ e \end{array}\right]+\left[\begin{array}{c} (N^{⊤}A_{c}R-N^{⊤}B_{c}K_{\zeta })\zeta \\ 0 \end{array}\right]$$

where $\bar{N}=\left[\begin{array}{cc} N & 0_{n\times n} \end{array}\right]$, and $E=\left[\begin{array}{cc} N & R \end{array}\right]$. Since the state matrix of the obtained closed-loop system is upper triangular, it is sufficient for matrices $\left(N^{⊤}A_{c}N-N^{⊤}B_{c}K_{z}\right)$ and $({\bar{N}}^{⊤}A_{c}-LC)E$ to be stable for the system to be stable. Condition (10) implies the stability of the first one, and condition (17) implies the stability of the second, as eigenvalues of matrix are equal to those of its transpose. □

Theorem 1 gives Separation Principle for LTI systems with explicit constraints in subspace representation. The proof follows the same principle as the proof in the analogous theorem for LTI systems.

**Figure 3:**
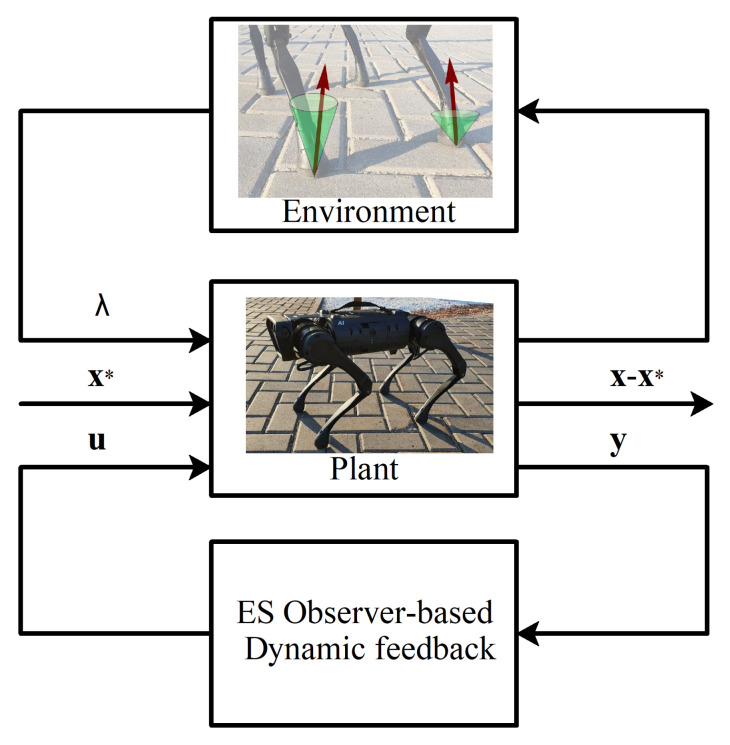
An illustration of the proposed control scheme; x and $x^{*}$ are actual and the desired value of the state of the robot, u is the control input, y is the measured output, and λ are reaction forces, due to the interaction with the environment.

A proposed state observer can be seen as a combination of a state observer in variable z and disturbance observer in variable ζ; indeed, a disturbance observer is an instrument for determining unknown constant components of the dynamics equations for linear systems, and ζ acts like one. A diagram of the proposed method as a Dynamic Output Feedback controller is shown in Figure 3.

While observer (16) works for a large class of linear systems, we can show that there are LTI systems for which it would not work, as will be demonstrated in Section 9. However, a different parameterization of state variables would allow us to construct a lower-dimensional stabilizable observer. This is the subject of the next section.

## 6. State Constraints in Observer Design

System (5) has its constraints expressed in terms of state derivative $\dot{x}$. However, in a number of cases, these constraints on $\dot{x}$ imply a constraint on x. For example, for mechanical systems, generalized velocities appear both in $\dot{x}$ and x, and some of the constraints are placed only on generalized velocities; thus, an equivalent constraint on x can be formed. Let us denote this *state constraint* as:

(21) $$G_{x}x=0,$$

where $G_{x}$ is a state constraint matrix. The intersection of the row space of $G_{x}$ and the span of R represent the space of static state values that are necessarily equal to zero and therefore do not need to be estimated. Therefore, all static state values that require estimation lie in the intersection of the null space of $G_{x}$ and the span of R. We will call orthonormal basis in this intersection $R_{SC}$. Remembering that N is the orthogonal complement of R and that intersection of subspaces can be found as the orthogonal complement of the union of their respective orthogonal complements, we produce the following formula for $R_{SC}$:

(22) $$R_{SC}={\left[\begin{array}{cc} \mathrm{col}\left(G_{x}^{⊤}\right) & N \end{array}\right]}^{⊥},$$

where operator ${\left(\cdot \right)}^{⊥}$ returns an orthonormal basis in the left null space of the matrix, or, equivalently, an orthonormal basis in the orthogonal compliment of the range (column space) of the operator.

**Example** **2.** *For mechanical systems with state x and constraint matrix $G=\left[\begin{array}{cc} F_{n} & 0\\ {\dot{F}}_{n} & F_{n} \end{array}\right]$ defined as in the example 1, the state constraint matrix $G_{x}$ takes the form: $G_{x}=\left[\begin{array}{cc} 0 & F_{n} \end{array}\right]$.*

Defining $\zeta =R_{SC}^{⊤}x$ and replacing row-space basis R with new basis $R_{SC}$ in the proposed observer design, we obtain SC observer (where SC stands for State Constraint):

(23) $$\left[\begin{array}{c} \dot{\hat{z}}\\ \dot{\hat{\zeta }} \end{array}\right]=\left[\begin{array}{cc} N^{⊤}A_{c}N & N^{⊤}A_{c}R_{SC}\\ 0 & 0 \end{array}\right]\left[\begin{array}{c} \hat{z}\\ \hat{\zeta } \end{array}\right]+\left[\begin{array}{c} N^{⊤}B_{c}u\\ 0 \end{array}\right]+L\left(y-C\left[\begin{array}{cc} N & R_{SC} \end{array}\right]\left[\begin{array}{c} \hat{z}\\ \hat{\zeta } \end{array}\right]\right)$$

To stabilize the SC observer, we find gains L, such that the following condition holds:

(24) $$\left[\begin{array}{c} N^{⊤}\\ R_{SC}^{⊤} \end{array}\right]\left(\left[\begin{array}{c} A_{c}^{⊤}N\\ 0 \end{array}\right]-C^{⊤}L^{⊤}\right)\in H$$

Both R and $R_{SC}$ are orthonormal; $\mathrm{rank}\left(R_{SC}\right)\leq \mathrm{rank}\left(R\right)$, meaning the former has the same number or fewer columns than the latter. The difference between projections of x on those two linear spaces is given by the following Theorem.

**Theorem** **2.** *For system (5), the only admissible value of the state x that lies in the range of R and is orthogonal to columns of $R_{SC}$ is x=0: $R_{SC}^{⊤}x=0\Leftrightarrow R^{⊤}x=0$.*

**Proof** **Theorem 2.** Since the span of $R_{SC}$ is the intersection of the span of R and null space of $G_{x}$, a vector $R^{⊤}x$, orthogonal to $R_{SC}$ has to lie in the row space of $G_{x}$, and according to (21) is equal to 0. Conversely, $\mathrm{col}\left(R_{SC}\right)\subset \mathrm{col}\left(R\right)$ implies that $R^{⊤}x=0\Rightarrow R_{SC}^{⊤}x=0$. □

Thus, the main difference between the observer (16) and (23) is that the latter has fewer state variables, making it easier to find gains L that satisfy the condition (24). In fact, it is possible to show systems where observer (16) cannot be stabilized because part of the state variables are not observed and cannot be inferred from the dynamics of the system, but have to be equal to zero due to the constraints. In particular, this is often true with unmeasured velocities for mechanical systems. Section 9 illustrates this.

## 7. The Effective States in Observer Design

From (9), we observe that the component of static state Rζ that lies in the null space of matrix $N^{⊤}A_{c}$ has no influence on the dynamics of the system. Hence, these states can be ignored from the control and observer design. Projecting row space of $N^{⊤}A_{c}$ onto the column space of $R_{SC}$, we find space of all values of $R_{SC}\zeta$ (and equivalently Rζ, according to Theorem 2) that influence control design $S_{ES}$, which we will call *effective row space* of the constrained system:

(25) $$R_{ES}=\mathrm{col}\left(R_{SC}R_{SC}^{⊤}A^{⊤}N\right),$$

where $R_{ES}$ is a basis in the effective row space of the constrained system.

Defining $\zeta =R_{ES}^{⊤}x$ and replacing row-space basis R with new basis $R_{ES}$ in the proposed observer design, we obtain ES observer (where ES stands for **E**ffective **S**tates):

(26) $$\left[\begin{array}{c} \dot{\hat{z}}\\ \dot{\hat{\zeta }} \end{array}\right]=\left[\begin{array}{cc} N^{⊤}A_{c}N & N^{⊤}A_{c}R_{ES}\\ 0 & 0 \end{array}\right]\left[\begin{array}{c} \hat{z}\\ \hat{\zeta } \end{array}\right]+\left[\begin{array}{c} N^{⊤}B_{c}u\\ 0 \end{array}\right]+L\left(y-C\left[\begin{array}{cc} N & R_{ES} \end{array}\right]\left[\begin{array}{c} \hat{z}\\ \hat{\zeta } \end{array}\right]\right)$$

To stabilize the ES observer, we find gains L, such that the following condition holds:

(27) $$\left[\begin{array}{c} N^{⊤}\\ R_{ES}^{⊤} \end{array}\right]\left(\left[\begin{array}{c} A_{c}^{⊤}N\\ 0 \end{array}\right]-C^{⊤}L^{⊤}\right)\in H$$

In Section 9, we will show that the proposed ES observer (26) requires fewer independent measurements (counted as the rank of the observation matrix C), compared with any other observer structure discussed in this paper.

## 8. Observer Gains Tuning

### 8.1. Compact Notation

Thus far, we have used an explicit notation, resulting in relatively long but clear expressions. It is in fact possible to make the notation more compact, by reintroducing matrices $\bar{N}=\left[\begin{array}{cc} N & 0_{n\times n^{*}} \end{array}\right]$, and $E=\left[\begin{array}{cc} N & R^{*} \end{array}\right]$, where $n^{*}$ equals the number of rows in E, and proposing to use a single variable $\hat{\chi }=\left[\begin{array}{c} \hat{z}\\ \hat{\zeta } \end{array}\right]$ to denote the state of the observer. Then, the expression for the observer becomes:

(28) $$\dot{\hat{\chi }}=\left({\bar{N}}^{⊤}A_{c}-LC\right)E\hat{\chi }+{\bar{N}}^{⊤}B_{c}u+Ly$$

In compact notation, if $R^{*}=R$ or $R^{*}=R_{SC}$, it follows that x=Eχ and, conversely, $\chi =E^{⊤}x$, where χ stands for the true value of $\hat{\chi }$. Control law then can be written as follows:

(29) $$u=-K\hat{\chi }$$

where $K=\left[\begin{array}{cc} K_{z} & K_{\zeta } \end{array}\right]$. Conditions (17), (24) and (27) can be written in compact notation as:

(30) $$E^{⊤}A_{c}^{⊤}\bar{N}-E^{⊤}C^{⊤}L^{⊤}\in H$$

This notation makes it obvious how the observer gains can be designed using standard techniques developed for the control of LTI systems, which will be shown in the next subsection.

### 8.2. Observer Gain Design

Using notation, introduced in the last subsection, we note that expression (30) is the closed-loop state matrix of the dual form of the observer error dynamics, and can be directly used to compute control gains using, for example, pole placement or LQR procedure. As a pseudo-code, it can be expressed as follows:

(31) $$L^{⊤}=\mathrm{place}\left(\left(E^{⊤}A_{c}^{⊤}\bar{N}\right),\;\;\left(E^{⊤}C^{⊤}\right),\;\;p\right)$$

where $p\in R^{n^{*}}$ is the vector of poles, desired for the closed loop system and place(·,·,·) is a pole placement algorithm, taking state matrix as the first input, control matrix as the second and desired poles as the third. Similarly, we can solve LQR problem:

(32) $$L^{⊤}=\mathrm{lqr}\left(\left(E^{⊤}A_{c}^{⊤}\bar{N}\right),\;\;\left(E^{⊤}C^{⊤}\right),\;\;Q,\;\;S\right)$$

where positive semidefinite $Q\in R^{n^{*}\times n^{*}}$ and positive definite $S\in R^{h\times h}$ are state error cost and observation correction cost matrices and lqr(·,·,·,·) is an lqr solver, taking state matrix as the first input, control matrix as the second, and cost matrices as the last two inputs.

## 9. Case Study: Table-Top Flywheel Inverted Pendulum

**Figure 4:**
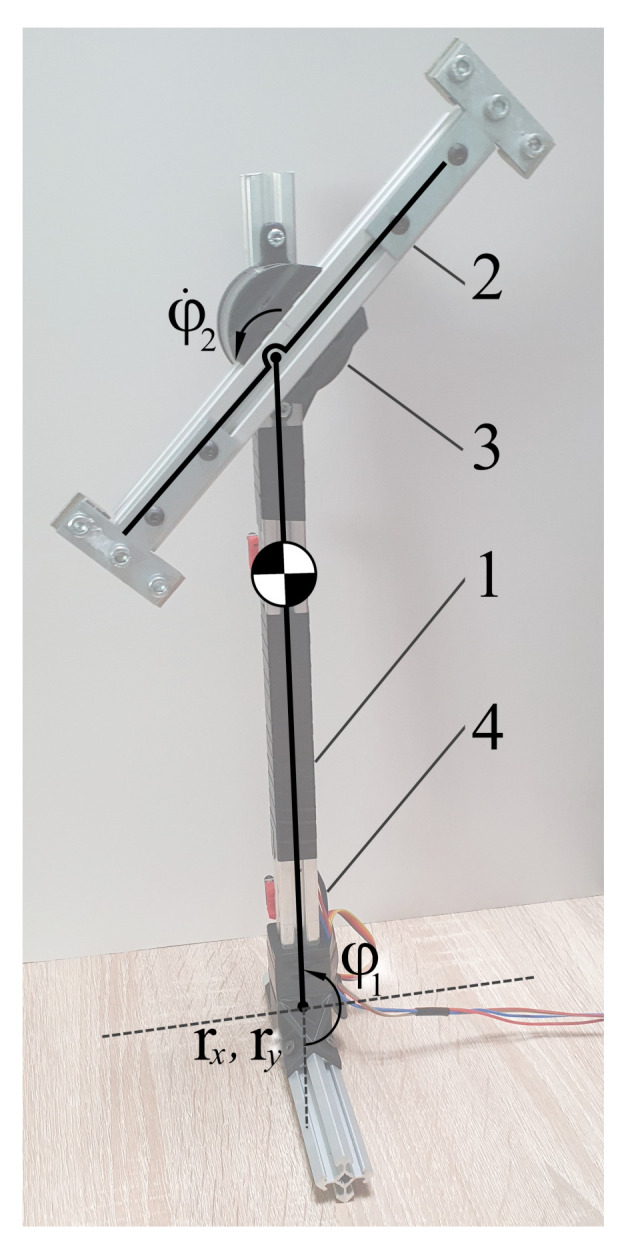
Table-top flywheel inverted pendulum: a schema (on the **left**) and a photo (on the **right**); 1—shaft, 2—load, 3—motor, 4—IMU module; in upper equilibrium $\varphi_{1}=\pi$.

Consider a planar flywheel inverted pendulum: an underactuated system consisting of a shaft connected to the ground via an un-actuated pin joint, and to a symmetrical load via a motor [44,45], as shown in Figure 4. The system can be described with four state variables: orientations of the shaft $\varphi_{1}$ and the load $\varphi_{2}$, and the rates of change of those orientations ${\dot{\varphi }}_{1}$, ${\dot{\varphi }}_{2}$. However, following floating-base dynamics formalism, actively used in bipedal robotics, we can also describe the system using eight state variables, adding the position of the tip of the shaft $r_{x}$, $r_{y}$ (2 variables for the planar case) and their rates of change ${\dot{r}}_{x}$, ${\dot{r}}_{y}$. This representation is convenient when the tip of the shaft can move along the supporting surface or leave it altogether, for example, due to reaction forces being limited to the friction cone. We will refer to this system as a table-top flywheel inverted pendulum.

A nonlinear model of the table-top flywheel inverted pendulum has the following general form:

(33) $$\left\{\begin{array}{c} H\ddot{q}+c=Tu+F^{⊤}\lambda \\ F\ddot{q}+\dot{F}\dot{q}=0 \end{array}\right.$$

where $q={\left[r_{x},r_{y},\varphi_{1},\varphi_{2}\right]}^{⊤}$, *u* is the control input (current applied to the motor), λ is the vector of reaction forces, is a vector of generalized coordinates, H is a generalized inertia matrix, c is a sum of generalized inertial, gravitational and dissipative forces, T is the control input Jacobian, and F is the reaction force Jacobian. The values those quantities have are presented below, given here up to two significant figures, values taken from least-squares-based parameter estimation; constant coefficients in the expressions below have appropriate units, not shown for conciseness (e.g., the coefficient 0.67 in (34) has units of kg, and 0.0017—units of $\mathrm{kg}\cdot m^{2}$):

(34) $$H=\left[\begin{array}{cccc} 0.67 & 0 & -0.21\cos(\varphi_{1}) & 0\\ 0 & 0.67 & -0.21\sin(\varphi_{1}) & 0\\ -0.21\cos(\varphi_{1}) & -0.21\sin(\varphi_{1}) & 0.071 & -0.0017\\ 0 & 0 & -0.0017 & 0.0017 \end{array}\right],$$

(35) $$c=\left[\begin{array}{c} 0.21{\dot{\varphi_{1}}}^{2}\sin\left(\varphi_{1}\right)\\ -0.21\cos\left(\varphi_{1}\right){\dot{\varphi_{1}}}^{2}+6.6\\ -2.1\sin(\varphi_{1})\\ 1.5\cdot 10^{-4}\dot{\varphi_{2}} \end{array}\right],\;\;F^{⊤}=\left[\begin{array}{cc} 1 & 0\\ 0 & 1\\ 0 & 0\\ 0 & 0 \end{array}\right],\;\;T=\left[\begin{array}{c} 0\\ 0\\ 0\\ 0.061 \end{array}\right]$$

One of the big advantages of this system for our purposes is the simplicity its dynamics attains when the equations (33) are solved with respect to the highest order derivatives:

(36) $$\left\{\begin{array}{c} {\ddot{r}}_{x}=0\\ {\ddot{r}}_{y}=0\\ {\ddot{\varphi }}_{1}=0.88u-0.0022\dot{\varphi_{2}}+29.6\sin\left(\varphi_{1}\right)\\ {\ddot{\varphi }}_{2}=36.8u-0.09\dot{\varphi_{2}}+29.6\sin\left(\varphi_{1}\right) \end{array}\right.$$

We can linearize the model around the unstable equilibrium, obtaining the following values for the state, control, and constraint matrices:

(37) $$A_{c}=\left[\begin{array}{cccccccc} 0 & 0 & 0 & 0 & 1 & 0 & 0 & 0\\ 0 & 0 & 0 & 0 & 0 & 1 & 0 & 0\\ 0 & 0 & 0 & 0 & 0 & 0 & 1 & 0\\ 0 & 0 & 0 & 0 & 0 & 0 & 0 & 1\\ 0 & 0 & 0 & 0 & 0 & 0 & 0 & 0\\ 0 & 0 & 0 & 0 & 0 & 0 & 0 & 0\\ 0 & 0 & 29.6 & 0 & 0 & 0 & 0 & -2.2\times 10^{-3}\\ 0 & 0 & 29.6 & 0 & 0 & 0 & 0 & -0.09 \end{array}\right],\;\;B_{c}=\left[\begin{array}{c} 0\\ 0\\ 0\\ 0\\ 0\\ 0\\ 0.88\\ 36.8 \end{array}\right]$$

Let us consider five cases, related to the availability of sensors: (1) when all states are measured (note that an observer is not actually needed in this case), (2) when all states except ${\dot{\varphi }}_{1}$ and ${\dot{\varphi }}_{2}$ are measured, (3) when all states except ${\dot{r}}_{x}$ and ${\dot{r}}_{y}$ are measured, (4) when only $\varphi_{1}$, $\varphi_{2}$, ${\dot{\varphi }}_{1}$ and ${\dot{\varphi }}_{2}$ are measured, and (5) when only $\varphi_{1}$ and $\varphi_{2}$ are measured. In all of these cases, matrix $E^{⊤}A^{⊤}\bar{N}$ has the form:

(38) $$E^{⊤}A^{⊤}\bar{N}=\left[\begin{array}{cc} \begin{array}{cccc} 0 & 0 & 29.6 & 29.6\\ 0 & 0 & 0 & 0\\ 1 & 0 & 0 & 0\\ 0 & 1 & -2.2\times 10^{-3} & -0.09 \end{array}\; & 0_{z\times (n^{*}-z)}\\ 0_{(n^{*}-z)\times z} & 0_{\left(n^{*}-z\right)\times \left(n^{*}-z\right)} \end{array}\right]$$

where z=4 is the number of columns of N (number of independent non-static states). The value of $n^{*}$ depends on the choice of the observer. For observer (16) $n^{*}=8$, for SC observer (23) $n^{*}=6$ and for ES observer (26) $n^{*}=4$. Matrix $\left(E^{⊤}C^{⊤}\right)$ is orthonormal; moreover, its elements are either 0 or 1. For conciseness, we only mention the rank of this matrix for each considered case. This information is found in Table 1.

Table 1 shows which of the observers (16), (23) and (26) is stabilizable for which measurement availability scenario. We also show which case allows the corresponding descriptor system to be stabilizable (using the definition from [1]).

As we can see from the Table 1, both the observer (16) and (23) work when all position variables and velocities ${\dot{r}}_{x}$${\dot{r}}_{y}$ are measured; it also satisfies the observably criterion for descriptor systems [1]. However, if instead of ${\dot{r}}_{x}$${\dot{r}}_{y}$ we measure ${\dot{\varphi }}_{1}$, ${\dot{\varphi }}_{2}$, the observer (16) no longer can be stabilized (and the observably criterion for descriptor systems would not be satisfied in this case either), while SC observer (23) and ES observer (26) both can be stabilized, being lower-dimensional systems. Moreover, the SC observer cannot be stabilized when $r_{x}$ and $r_{y}$ are not measured directly. The ES observer, on the other hand, excludes those from the list of estimated variables, as they do not affect the dynamics of the system (as discussed in Section 7); thus, it can be stabilized without measuring these states.

**Figure 5:**
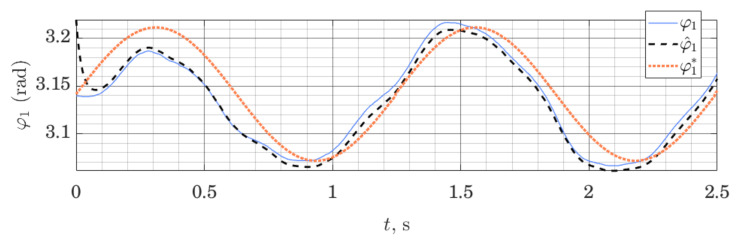
Orientation of the shaft: measured $\varphi_{1}$, estimated ${\hat{\varphi }}_{1}$ and linearization point $\varphi_{1}^{*}$; performance of the ES Observer on the table-top flywheel inverted pendulum, around the upper equilibrium point.

**Figure 6:**
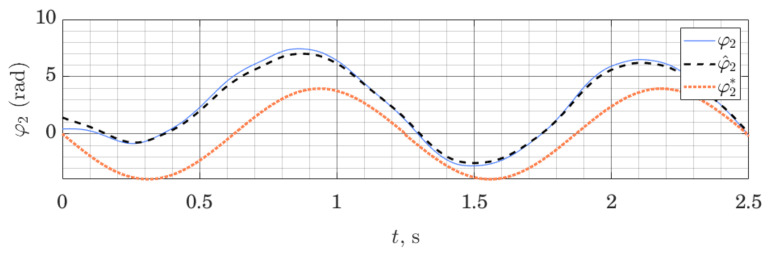
Load orientation: measured $\varphi_{2}$, estimated ${\hat{\varphi }}_{2}$ and linearization point $\varphi_{2}^{*}$; performance of the ES Observer on the table-top flywheel inverted pendulum, around upper equilibrium point.

We can show that the method works not only on the LTI systems (as is proven by stability analysis), but can also be employed on nonlinear systems, linearized around a given trajectory. Figure 5 and Figure 6 show behavior of the ES Observer used on the table-top flywheel inverted pendulum discussed in this section. The mechanism follows a cyclic trajectory around the upper unstable equilibrium point. The motion of the system (shown as a blue graph on all figures) is placed far from the nominal trajectory, used for linearization (shown as a dotted orange graph), affecting the accuracy of the model used by the observer. The experimental setup was designed to allow two types of measurements: (1) change in orientation of both links are measured with a 12-bit magnetic encoder (with the resolution of 0.088 degrees), (2) change in orientation of the load is measured with a 12-bit magnetic encoder, while the orientation of the shaft is measured with the use of two IMU modules MPU6050, whose readings from gyroscopes and accelerometers are fused to compute an estimate of the link’s orientation.

We can see that the observer drives down the initial estimation error. Since the motion of the system was taking place far from the linearization trajectory, the model available to the observer was not accurate; together with limited sensor accuracy, this illustrates a typical use-case for observers in mobile robotics, where the availability of high-quality sensors and exact models are limited, while exact tracking of a trajectory is not always possible or necessary. With this experiment, we wanted to demonstrate that these circumstances do not prevent the use of the proposed Observer, subject to proper tuning. For experiments shown in Figure 5 and Figure 6, the tuning method (32) was used, with matrices Q=diag(100,100,10,10) and S=diag(0.05,1). This, however, is only a qualitative result, showing the possibility of using such observers. The study of the analytic conditions guaranteeing stability of the proposed observers for nonlinear systems in the vicinity of the linearization point would be of interest.

**Figure 7:**
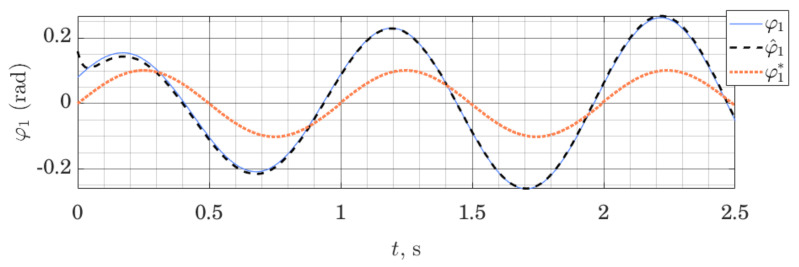
Orientation of the shaft: measured $\varphi_{1}$, estimated ${\hat{\varphi }}_{1}$ and nominal trajectory $\varphi_{1}^{*}$; performance of the ES Observer on the table-top flywheel inverted pendulum, around lower equilibrium point.

**Figure 8:**
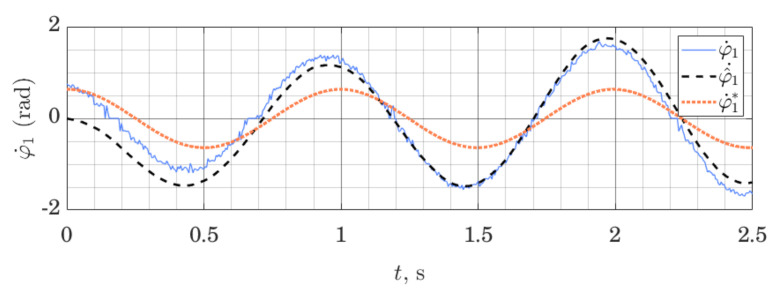
Angular velocity of the Shaft: measured ${\dot{\varphi }}_{1}$, estimated ${\dot{\hat{\varphi }}}_{1}$ and linearization point ${\dot{\varphi }}_{1}^{*}$; Performance of the ES Observer on the table-top flywheel inverted pendulum, around a lower equilibrium point.

Figure 7 and Figure 8 show the behavior of the ES observer when used for the same system, but this time around its lower equilibrium point. Studying Figure 7 and Figure 8, we note that initial estimation error quickly tends to zero both for the position and the velocity variables. However, the velocity variable, which is not measured directly and relies on the imprecise dynamics model to be estimated, is not tracked perfectly. These experiments were performed using the same observer gains as previously.

## 10. Case Study: Quadruped Robot

In this section, we explore the influence contact scenarios have on the performance and structure of the proposed observer, using as an example an actual quadruped robot Unitree A1. As was highlighted previously, proposed SC and ES observers have equal or lower-dimensional state space compared with the original robot described in floating base coordinates (joint angles and position and orientation of the trunk), but an equal or higher number of coordinates than the same robot described in minimal coordinate representation. In this section, we demonstrate a number of contact scenarios where values of the static part of the state space $R^{*}\zeta$ are needed to solve inverse dynamics (or, equivalently, when $R_{ES}$ has a non-zero number of columns).

**Figure 9:**
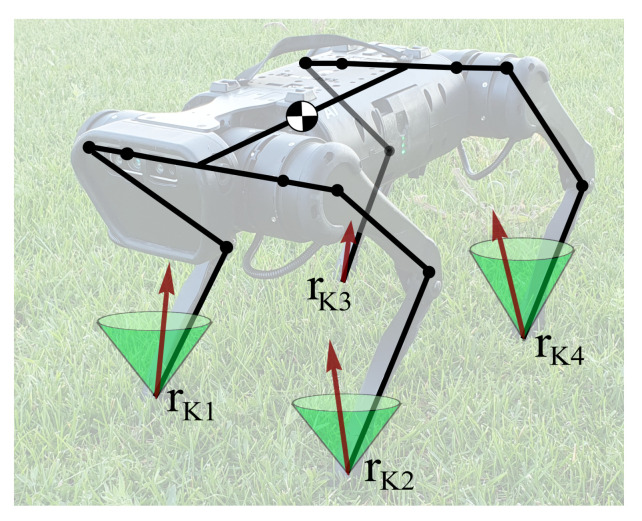
Quadruped robot Unitree A1 in a stance: all feet are in contact with the ground in points $r_{Ki}$, red arrows indicate contact forces, and green cones illustrate friction cones; no slipping is assumed—contact forces lie inside friction cones; $r_{Oi}$ are knee joint positions.

Figure 9 shows a scheme of the Unitree A1 quadruped with four points of contact with the ground $r_{Ki}$, with contact forces lying in the interior of the respective friction cones: we do not consider scenarios, where reaction force lies on the boundary of the friction cone, and the constraint is violated by slipping.

A robot’s leg consists of a hip link connected to the trunk via two motors and a shin link connected to the hip link via a single motor, installed at the hinge $r_{Oi}$; we will refer to the later points as “knees”. Contact points $r_{Ki}$ lie on the contact pads installed on the shin links. In the following, we will consider two types of constraints: (1) constraints on the position of contact points $r_{Ki}=\mathrm{const}$, arising from the foot being in contact with the environment, and (2) constraints on the positions of the knee hinges $r_{Oi}=\mathrm{const}$, arising from accidental contacts with the environment, for example during blind stair ascent or descent, or from the robot attempting to "stay on its knees" for vertical stability.

Table 2 shows ranks (which is equivalent to the number of columns, as these matrices are orthonormal) of matrices R, $R_{SC}$ and $R_{ES}$, which define the number of static state components that are required to be observed for the full state observer (16), SC observer (23) and ES observer (26) respectively, in different contact scenarios.

We can note that the first contact scenario in Table 2 is the stance on all legs, and the second is the stance on only three legs. In both cases, the number of static states that require estimation is half of the total number of static states, which is the effect of state constraints and the use of an SC observer. The use of ES Observer does not produce a benefit here. The third scenario in Table 2 represents the robot standing on its rear shins, with its front legs free for manipulation tasks. Here, the use of ES observer allows us to drop one or two dimensions from the observer state, depending on the robot configuration. The last case represents the robot standing on three feet, while the last one (the rear left one) contacts the environment via its knee, which can happen during staircase descent. Here, again, the ES observer obtained one or two dimensions fewer than the SC observer, and 13–14 dimensions fewer than a full state observer, showing a clear benefit of using this Observer design.

## 11. Case Study: Motion of a Quadruped in the Sagittal Plane

In this section, we demonstrate the performance of the proposed observer on a quadruped robot moving in the sagittal plane. Following [46], we represent it as a five-link structure. This allows us to describe the robot’s position with seven generalized coordinates: the position and orientation of the robot’s trunk and its four joint angles. The flat model pairs up the legs—the configuration of both front legs is described by the same two coordinates; the same is true about both rear legs. We denote as $\phi_{1}$ and $\phi_{2}$ angles associated with the joints connecting the front and rear legs, respectively, and $\phi_{3}$ and $\phi_{4}$ are angles in the knee joints in the front and rear legs. Parameters of the robot used in the simulation are shown in Table 3.

The simulation is set up as follows. The model of the robot is linearized along a nominal trajectory, and stabilizing affine control law is found for the resulting linear model at each time step by solving the Riccati equation. In the same way, observer gains are found at each time step. That represents a gain scheduling approach for control and observer design [47]. We log both the evolution of the state of the robot and the state of the observer; for conciseness, we only present the evolution of the observer error with respect to joint angles $\phi_{i}$ and their estimates ${\hat{\phi }}_{i}$.

**Figure 10:**
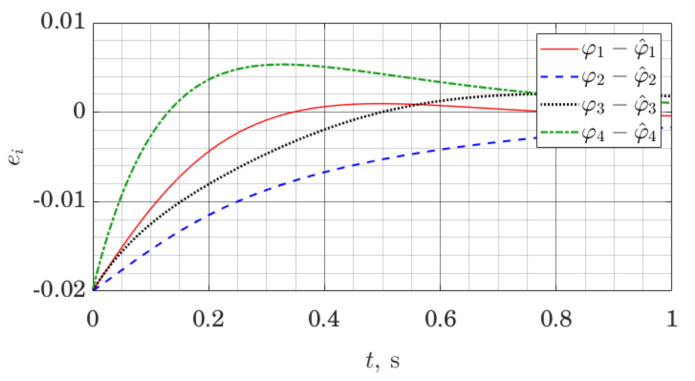
Joint angle estimation error for a five link robot with the ES Observer.

**Figure 11:**
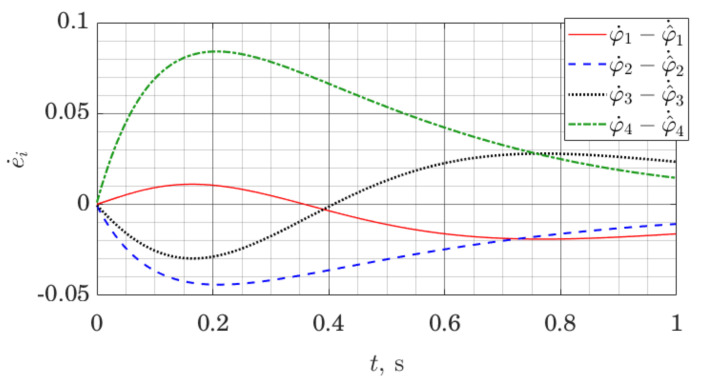
Joint angle rates estimation error for a five link robot with the ES Observer.

Figure 10 and Figure 11 demonstrate observer error dynamics when we measure joint angles $\phi_{i}$, as well as orientation of the trunk, as well as the rate of change of that orientation. In Figure 10 and Figure 11, $e_{i}=\phi_{i}-{\hat{\phi }}_{i}$.

Let us note that observer error graphs on both Figure 10 and Figure 11 demonstrate convergence to zero; however, as a numerical result, this only suggests practical applicability of the methods. Formal stability guarantees for the closed-loop nonlinear system using this observer would have to be given in a similar manner as for other gain scheduling approaches [47].

## 12. Comparative Analysis of the Effective States Observer and Extended Kalman Filter

It is of practical interest to better understand the relations of the proposed observer and the existing state estimation methods. A broad outline of these relations was given in Section 2.2 and illustrated in Figure 1. Here, we illustrate the relation between the proposed ES observer and Extended Kalman Filter (EKF) [48,49,50], as one of the most widely used state estimation methods.

We implement both ES Observer and EKF on the flat quadruped model, presented in the previous section, with the same dynamics and measurement model, but with added noise. We model process noise and sensor noise as random signals with covariance matrices $Q_{k}=9\times 10^{-8}I$ and $R_{k}=10^{-4}I$, respectively. The ES Observer is tuned using LQR procedure (32), with cost matrices $Q=10^{3}I$ and S=I. Note that the ES filter does not account for noise, and it also uses a linear model rather than the nonlinear dynamics available to EKF. At the same time, EKF is not designed to take advantage of the structure of the constraints.

To access the quality of the observers, we introduce a quality criterion as a cost function $J_{c}$:

(39) $$J_{c}=\sum_{i}\left(\left|\left|x_{i}-{\hat{x}}_{i}\right|\right|\Delta t_{i}\right)$$

where $x_{i}$ is the true value of the state on the *i*-th time step, ${\hat{x}}_{i}$ is the estimated value of the state, and $\Delta t_{i}$ is the length of the *i*-th time step.

Using a quality criterion (39), we can simulate the behavior of ES Observer and EKF from the same initial conditions, and compare the results. Since both are model-based methods and rely on the exact model information, we perform two experiments: (1) where the initial state of the system lies near the nominal trajectory and (2) where the initial state of the system lies far from it. The initial state of the system is denoted as $x_{0}$ and the initial point on the nominal trajectory is given as $x_{0}^{*}$. Table 4 shows the simulation result.

As we can see, near the nominal trajectory EKF demonstrates a much smaller cost than the ES Observer, which should be expected since it had access to the exact nonlinear model of the system and ES Observer did not have access to the information about noise. However, further from the nominal trajectory, EKF’s performance decreases much faster than that of the ES Observer.

We should note that the ES Observer is designed for systems without either process or sensor noise; at the same time, EKF is designed for systems without explicit constraints. That limits the conclusions that can be inferred from the results presented here. One of the main conclusions that have to be drawn is that gain design for ES Observer should be studied further, focusing on the inclusion of different optimality criteria in the gain design. This is a possible path because ES Observer imposes the observer structure, but not the design method for the observer gains.

## 13. Application of the Proposed Method

As has been stated previously, the main field where the proposed observer design method can be applied is state estimation of robots with explicit constraints. These include walking robots of various kind, and especially new types of robots for which the platform-specific observers have not yet been developed. The motivation here is the simplicity of the observer structure and the ease of gain design.

The second potential application is benchmarking other observers, including platform-specific ones. This is again linked with the ease of implementation of the proposed observer.

Finally, but possibly the most interesting area of application, is sensor distribution design. Since the proposed observer automatically finds minimal dimension of the space of the static state variables in each contact situation, it can be used to assess which sensors (and which sensor placement) would be sufficient to extract all necessary information about the state of the robot. This might be of practical value for rapid design and prototyping of new types of legged robots and other contact-driven mobile platforms. If the robot is designed to perform motions that are known in advance, and the contact interaction scenarios are also known, the ability to stabilize the ES observer can be used as a criterion in sensor placement decisions.

## 14. Conclusions

In this work, a novel observer design for linear systems with explicit constraints has been proposed. The proposed observer takes advantage of the structure of the constraints equations, making it possible to either recover a minimal coordinate representation or a representation with fewer coordinates (compared to, e.g., floating base representation for walking robots), making lower requirements on the measured outputs (hence, requiring fewer sensors) needed to make the observer stabilizable while keeping the observer state sufficient for both the feedback and feed-forward control laws design. The paper showed the observer structure, stability condition, gain design method, and demonstrated separation principle for the proposed observer.

The observer was tested on the table-top flywheel inverted pendulum (a hardware experiment), a Unitree A1 quadruped robot in a number of contact scenarios, as well as a quadruped robot model moving in the sagittal plane (where simulation results were demonstrated). The paper showed that the observer recovers minimal coordinate representation for a table-top flywheel inverted pendulum. Using Unitree A1 as an example, we showed that the observer automatically takes advantage of the given contact interaction scenario, lowering its state-space dimensions when possible. Simulations with a quadruped robot model moving in the sagittal plane demonstrated the convergence of the joint angle estimation errors and joint angle rate of change estimation errors to zero. Comparative analysis with Extended Kalman Filter showed that the proposed observer can perform better than EKF for a system with explicit constraints when the motion takes place further from the nominal trajectory, even in the presence of process and sensor noise; however, when the motion takes place on the nominal trajectory, EKF performs better, as the proposed observer gain design does not take into account the properties of un-modelled random inputs.

The proposed observer can be used in control design for linear systems with explicit constraints, or in gain-scheduling methods, where a nonlinear model is replaced with its local linearizations. The method fills the gap in orthogonal projection-based control design methods with respect to dynamic output feedback design. It requires the use of SVD decomposition and stabilizing feedback design algorithms (such as LQR or pole-placement), and is platform-independent, which makes it interesting in practical applications. The questions of stability verification for the closed-loop dynamics of nonlinear systems using the proposed observer are a subject of further study. The questions related to optimal gain design for such observers with respect to various metrics can be of further interest.

## Figures and Tables

**Table 1:** Analysis results for different observer types.

States Measured	Rank C	Observer Stabilizable			Descriptor Observable
		Full State (16)	SC (23)	ES (26)	
all	8	yes	yes	yes	yes
$\varphi_{1}$, $\varphi_{2}$, $r_{x}$, $r_{y}$, ${\dot{r}}_{x}$, ${\dot{r}}_{y}$	6	yes	yes	yes	yes
$\varphi_{1}$, $\varphi_{2}$, $r_{x}$, $r_{y}$, ${\dot{\varphi }}_{1}$, ${\dot{\varphi }}_{2}$	6	no	yes	yes	no
$\varphi_{1}$, $\varphi_{2}$, ${\dot{\varphi }}_{1}$, ${\dot{\varphi }}_{2}$	4	no	no	yes	no
$\varphi_{1},\varphi_{2}$	2	no	no	yes	no

**Table 2:** Analysis results for different observer types.

#	Constrained (Constant) Values	Rank		
		R	$R_{\mathrm{SC}}$	$R_{\mathrm{ES}}$
1	$r_{K1}$, $r_{K2}$, $r_{K3}$, $r_{K4}$	24	12	12
2	$r_{K1}$, $r_{K2}$, $r_{K3}$	18	9	9
3	$r_{K3}$, $r_{K4}$, $r_{O3}$, $r_{O4}$	20	10	8–9
4	$r_{K1}$, $r_{K2}$, $r_{K3}$, $r_{O4}$	24	12	10–11

**Table 3:** Parameters of the flat quadruped.

Link	Parameters	
	Mass, kg	Length, m
Trunk	10	0.5
Front Thigh	2	0.3
Rear Thigh	2	0.3
Front Shin	2	0.3
Rear Shin	2	0.3

**Table 4:** Comparison results for ES Observer and Extended Kalman Filter on a flat quadruped robot.

Observer	Cost $J_{c}$	
	$\left|\left|x_{0}-x_{0}^{*}\right|\right|$ = 0.0027	$\left|\left|x_{0}-x_{0}^{*}\right|\right|$ = 0.027
Extended Kalman Filter	0.0440	1.0171
Effective States Obsever	1.0171	1.3714

## Abbreviations

The following abbreviations are used in this manuscript:

DAE	Differential-Algebraic Equations
ODE	Ordinary Differential Equations
LQR	Linear-Quadratic Regulator
LTI	Linear Time-Invariant
MPC	Model-Predictive Control
SVD	Singular Value Decomposition
IMU	Inertial Measurement Unit
EKF	Extended Kalman Filter
